# Enhancing brain tumor detection in MRI images using YOLO-NeuroBoost model

**DOI:** 10.3389/fneur.2024.1445882

**Published:** 2024-08-22

**Authors:** Aruna Chen, Da Lin, Qiqi Gao

**Affiliations:** ^1^College of Mathematics Science, Inner Mongolia Normal University, Hohhot, China; ^2^Center for Applied Mathematical Science, Inner Mongolia, Hohhot, China; ^3^Laboratory of Infinite-Dimensional Hamiltonian System and Its Algorithm Application, Ministry of Education (IMNU), Hohhot, China; ^4^School of Mathematical Sciences, Inner Mongolia University, Hohhot, China

**Keywords:** brain tumors, target detection, YOLOv8, Inner-GIoU, CBAM

## Abstract

Brain tumors are diseases characterized by abnormal cell growth within or around brain tissues, including various types such as benign and malignant tumors. However, there is currently a lack of early detection and precise localization of brain tumors in MRI images, posing challenges to diagnosis and treatment. In this context, achieving accurate target detection of brain tumors in MRI images becomes particularly important as it can improve the timeliness of diagnosis and the effectiveness of treatment. To address this challenge, we propose a novel approach–the YOLO-NeuroBoost model. This model combines the improved YOLOv8 algorithm with several innovative techniques, including dynamic convolution KernelWarehouse, attention mechanism CBAM (Convolutional Block Attention Module), and Inner-GIoU loss function. Our experimental results demonstrate that our method achieves mAP scores of 99.48 and 97.71 on the Br35H dataset and the open-source Roboflow dataset, respectively, indicating the high accuracy and efficiency of this method in detecting brain tumors in MRI images. This research holds significant importance for improving early diagnosis and treatment of brain tumors and provides new possibilities for the development of the medical image analysis field.

## 1 Introduction

Brain tumors refer to the abnormal proliferation of cells within or surrounding the brain, manifesting either as benign or malignant diseases (1–3). Benign tumors, such as meningiomas, typically grow slowly and do not invade surrounding tissues; whereas malignant tumors, such as glioblastomas, usually grow rapidly, are highly invasive, and are difficult to completely remove. Brain tumors directly compress brain functions, leading to increased intracranial pressure and symptoms such as headaches, blurred vision, and changes in cognition and mood (4–6). If the tumor persists or worsens, it may cause permanent neurological damage or fatal outcomes. Moreover, depending on the type and location of the tumor, patients may experience a range of health issues from mild memory loss to severe physical disabilities. For example, gliomas are the most common type of malignant brain tumor in adults, originating from glial cells in the brain and spinal cord; while medulloblastomas, more common in children, typically occur in the cerebellum, affecting balance and coordination. Each type of brain tumor has its unique biological characteristics, treatment responses, and prognoses.

In terms of treatment, the management of brain tumors typically requires a combination of multiple therapeutic strategies. Surgery aims to remove as much of the tumor tissue as possible, while radiation and chemotherapy are used to eliminate tiny residual lesions or control further growth of the tumor (7–9). Early detection and precise localization are particularly important for the diagnosis and treatment of brain tumors. In recent years, object detection technology applied in the field of medical imaging, especially in the automatic detection and localization of brain tumors, has provided significant technical support. These technologies use advanced image analysis and machine learning algorithms to identify abnormal structures from complex medical imaging data, greatly assisting physicians in making rapid and accurate diagnoses (10–12). This not only lays the foundation for targeted and immunotherapy for specific types of brain tumors but also offers patients more personalized and effective treatment options.

However, despite significant progress in object detection technology across various domains, its application in MRI image analysis still faces some noticeable limitations. Confronted with MRI images, current object detection algorithms primarily grapple with the challenge of handling the inherent high heterogeneity and complexity of these imaging techniques (13, 14). MRI images exhibit significant differences in contrast, spatial resolution, and presented anatomical details, posing challenges to the universality and accuracy of algorithms. Additionally, the quality and consistency of MRI images are influenced by various factors, including equipment configuration, imaging technique parameters, and patient movement during imaging (15). These factors may lead to increased noise and contrast issues in images, thereby affecting the performance of object detection algorithms. Moreover, due to the immense diversity in the morphology, size, and boundaries of brain tumors, existing algorithms often struggle to accurately differentiate between tumor tissue and normal brain tissue, especially in cases where tumor boundaries are unclear or contrast with surrounding tissue is low.

In recent years, with the rapid development of deep learning technology, various object detection algorithms have been widely applied to the automatic detection and localization of brain tumors. Among these, the Faster R-CNN algorithm uses its built-in Region Proposal Network (RPN) to automatically identify candidate regions in images, then refines the classification and bounding box regression of these regions, enhancing detection accuracy (16). However, Faster R-CNN has relatively slow processing speeds, which can limit its application in real-time scenarios. The YOLO algorithm is known for its rapid image processing capability, dividing images into multiple grids, each predicting bounding boxes and probabilities, facilitating fast detection (17). However, its accuracy decreases when handling medical images with complex backgrounds. SSD combines multi-scale feature maps to improve detection precision, suitable for tumors of various sizes, balancing speed and accuracy, though it still has room for improvement in detecting very small or vague tumors. RetinaNet uses focal loss to address class imbalance, optimizing the detection of hard-to-recognize tumors, though it requires substantial resources during training (18). Mask R-CNN, an extension of Faster R-CNN, not only detects targets but also generates high-quality segmentation masks, excelling in precisely delineating tumor and normal tissue boundaries but requiring significant computational resources (19). Additionally, the latest YOLOv8 stands out in enhancing processing speed and accuracy, especially suitable for real-time diagnostic environments (20), but it still needs further optimization for accurately detecting highly overlapping tumor regions and small tumors. The development of these technologies continues to advance the field of medical imaging, and despite some limitations, their role in future medical diagnostics is increasingly important.

To address the limitations of existing methods, this paper introduces the YOLO-NeuroBoost model. This model integrates multiple innovative technologies aimed at enhancing the detection of brain tumors in MRI images. Firstly, the model utilizes KernelWarehouse technology, dynamically selecting and assembling convolution kernels based on input data features, thereby replacing traditional convolution kernels and significantly enhancing the model's adaptability and overall performance. Secondly, by incorporating the CBAM, it strengthens the recognition and processing of crucial image features, effectively improving the accuracy of brain tumor detection. Lastly, the implementation of the Inner-GIoU loss function allows for more precise bounding box localization in complex image scenarios and enhances sensitivity to various sized targets through adjustments of the scaling factor ratio, further improving detection accuracy. Through the integration of these advanced technologies, the YOLO-NeuroBoost model provides a practical solution for accurate and reliable brain tumor detection, significantly aiding in early diagnosis and treatment planning for patients.

- The YOLO-NeuroBoost model is proposed, which significantly enhances the detection capability of brain tumors in MRI images by integrating innovative technologies such as KernelWarehouse, CBAM, and Inner-GIoU loss function.
- The methods proposed in this paper demonstrate strong practicality and versatility. They can not only be applied to the detection of brain tumors in MRI images but also extended to other medical imaging fields, providing new insights and methods for medical image analysis.
- Through the research presented in this paper, more accurate and reliable methods for brain tumor detection are provided for clinical medicine. This aids physicians in early diagnosis and formulation of more effective treatment plans, offering crucial support for patients with brain tumors.

Here is the structure of the remaining work. Section 2 introduces the related work in brain tumor object detection. Section 3 will elaborate on the principles of our approach. Section 4 will describe our experimental process. Finally, Section 5 summarizes and provides an outlook on future work.

## 2 Related work

### 2.1 Machine learning methods and their applications in brain tumor detection in MRI images

In the field of brain tumor detection and diagnosis, Magnetic Resonance Imaging (MRI) has become an indispensable tool, with machine learning methods playing a crucial role in this process (13). Various algorithms have been developed and applied for the automatic detection and identification of brain tumors from MRI images. For example, clustering algorithms based on Particle Swarm Optimization (PSO) effectively identify tumor regions by analyzing the centroids of numerous brain tumor patterns obtained from MRI images (21, 22). Additionally, Discrete Wavelet Transform (DWT) is widely used as an initial step in feature extraction, transforming input images to extract key information, followed by Principal Component Analysis (PCA) to reduce the dimensionality of the feature vectors, thereby simplifying subsequent processing steps (23). Support Vector Machines (SVM) are powerful classification tools commonly used to categorize MRI brain images into normal and abnormal categories (24). Moreover, Feedback Pulse Coupled Neural Networks (FPCNN) are also used for the segmentation and detection of Regions of Interest (ROI), which are crucial steps in determining the location of brain tumors. Simultaneously, DWT continues to play a role in the feature extraction stage, enhancing the analysis capability of the images (25). In more complex analyses, the Generalized Autoregressive Conditional Heteroskedasticity (GARCH) model is introduced to handle potential time-series data, which is particularly important in dynamic MRI analysis (26, 27). Genetic Algorithms (GA) combined with curve-fitting techniques and SVM further enhance the model's predictive accuracy and generalization capabilities (28). The MRI image processing workflow typically begins with image input, followed by image enhancement, skull stripping, fuzzy C-means clustering, and feature extraction, among other steps. These steps form a comprehensive preprocessing workflow that lays the foundation for subsequent machine learning analysis. Additionally, applications of Hybrid Fuzzy Segmentation-Self Organizing Map (HFS-SOM) clustering and Gray Level Co-occurrence Matrix provide effective means for extracting complex texture features from MRI images (28, 29). Lastly, methods combining Extended Kalman Filters (EKF) with Support Vector Machines (SVM) offer a novel perspective for MRI image analysis, dynamically updating and improving model parameters to adapt to complex and changing image data characteristics, thus enhancing the accuracy and efficiency of brain tumor detection (30). The integrated application of these methods demonstrates the powerful potential of advanced image processing and machine learning technologies in brain tumor detection and diagnosis.

### 2.2 Advances in brain tumor detection in MRI images using YOLO algorithm series

In the field of brain tumor detection in MRI images, the YOLO (You Only Look Once) series of algorithms has made significant advancements in accuracy, speed, and functionality through a series of version upgrades. Initially, YOLOv3, an early version, introduced multi-scale prediction and utilized the Darknet-53 backbone network to extract features at three different scales, effectively enhancing the recognition capabilities for brain tumors of varying sizes (1, 31). Despite its substantial improvements in detection speed and accuracy, YOLOv3 still had room for improvement in sensitivity to very small or blurred tumors. Subsequently, YOLOv4, while maintaining high speed, incorporated the CSPDarknet53 backbone network along with more data augmentation techniques and attention mechanisms, further optimizing the network's generalization ability (32). These enhancements not only improved YOLOv4's ability to capture details of brain tumors, especially in complex backgrounds but also enhanced the model's stability and accuracy. Moving forward, YOLOv5, with its more flexible and modular design, simplified the deployment and usage of the model. By adopting a smaller model size and automated hyperparameter optimization, YOLOv5 significantly reduced the demand on computing resources while maintaining high accuracy, making it exceptionally effective in real-time brain tumor detection applications (33). YOLOv6 and YOLOv7 continued to innovate in model architecture and training strategies, such as introducing a more advanced FPN structure for feature processing, which enhanced the model's capability to recognize complex forms of brain tumors, demonstrating the further potential of deep learning technologies (20, 27, 34). The latest YOLOv8 made significant design innovations in the backbone network and Neck sections, such as replacing the C3 structure from YOLOv5 with a more gradient-rich C2f structure, and optimizing the channel numbers for different scale models. In the Head section, by adopting a decoupled head structure (Decoupled-Head) and shifting from an Anchor-Based to an Anchor-Free design, combined with the use of the Task-Aligned Assigner for positive and negative sample matching and introducing the Distribution Focal Loss (DFL), YOLOv8 significantly enhanced the model's accuracy and efficiency in detecting brain tumors. Overall, the iterative and optimized development of the YOLO algorithms continues to push the frontiers of machine learning in medical image analysis, particularly in the detection of brain tumors in MRI images (20).

## 3 Method

### 3.1 Overview of our network

**Figure 1:**
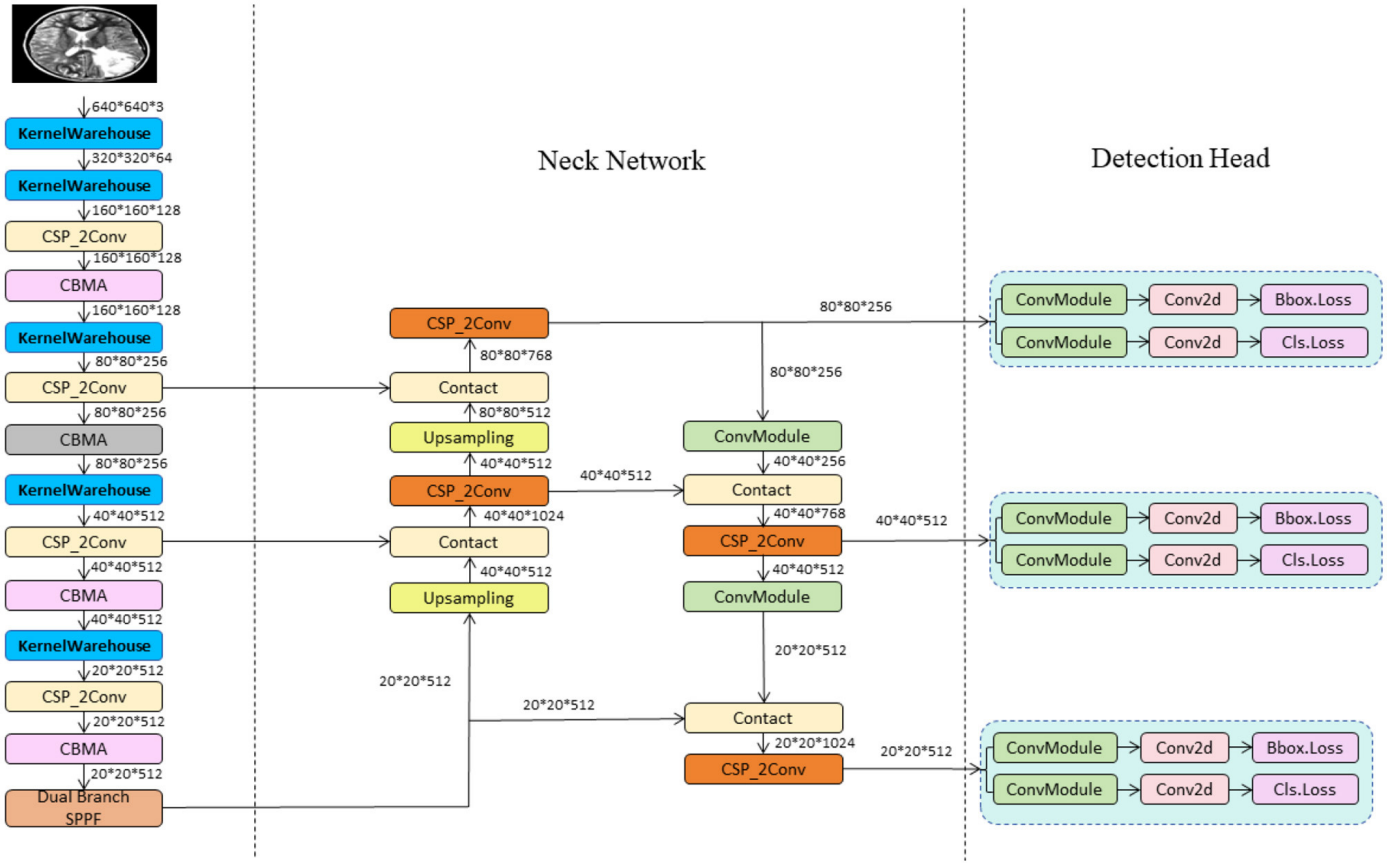
The diagram of the YOLO-NeuroBoost network structure.

In this study, we propose the YOLO-NeuroBoost model, specifically innovating upon the YOLOv8 algorithm to enhance the accuracy and efficiency of brain tumor detection in MRI images. Our approach includes several technical innovations: Firstly, we introduced the KernelWarehouse to replace traditional convolutional kernels. In this enhancement, we reevaluated the dependency relationships of convolutional parameters within and across layers, and redefined the fundamental concepts of “kernels,” “assembled kernels,” and “attention functions” within the context of dynamic convolution. KernelWarehouse, as a more generalized form of dynamic convolution, allows the model to dynamically select and assemble the most suitable convolutional kernels based on the characteristics of the input data, significantly improving the model's adaptability and performance. Secondly, to further enhance the model's ability to capture important features in images, we incorporated the CBAM (Convolutional Block Attention Module) attention mechanism. CBAM enhances the model's focus on critical areas of the image through spatial and channel attention sequences, thereby improving the precision of brain tumor detection. Lastly, we improved the loss function by introducing the Inner-GIoU loss. This new loss function assists in the precise localization of bounding boxes by calculating the IoU (Intersection over Union) loss, especially in complex or occluded scenarios. To adapt the loss function to different datasets and detection requirements, we introduced a scaling factor ratio that controls the size of the auxiliary bounding boxes used for loss calculation, adjusting the model's sensitivity to targets of varying sizes. These improvements not only enhance the accuracy of brain tumor detection but also optimize the model's versatility and robustness in processing different types of MRI image data. This series of technical innovations opens new possibilities for the application of deep learning in the field of medical image analysis. The network architecture of YOLO-NeuroBoost is shown in Figure 1.

### 3.2 YOLOv8 network

**Figure 2:**
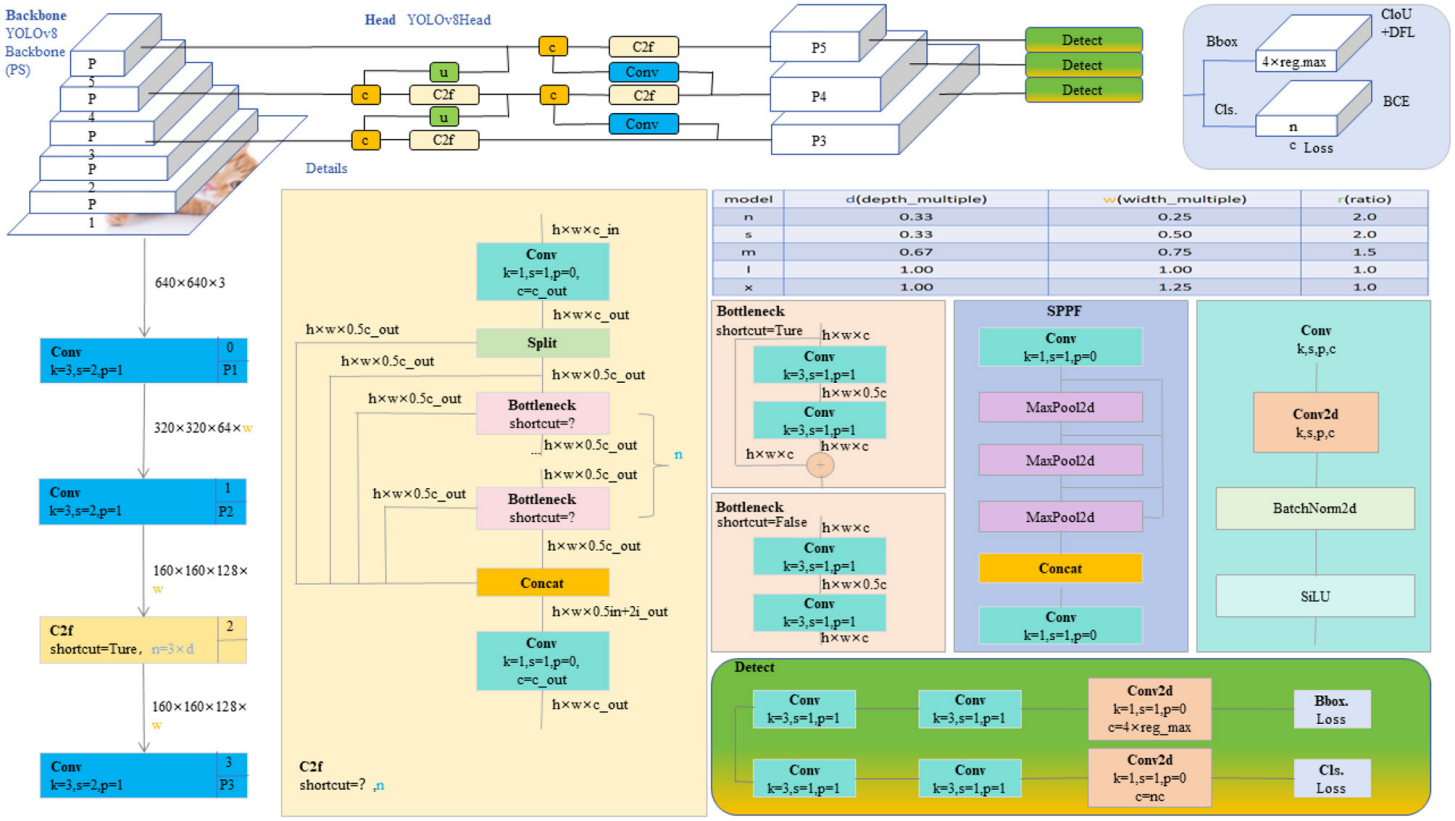
The diagram of the YOLOv8 network structure (20).

The YOLO model is a revolutionary object detection framework known for its single-stage detection mechanism, which achieves real-time performance while maintaining high accuracy. YOLOv8, as the latest iteration in the series, continues to use and improve upon the design concepts of previous versions (35). This version features a more refined backbone network and Neck part, inspired by the ELAN design of YOLOv7, where the C3 module from YOLOv5 has been replaced with the C2f module, which provides richer gradient flow, enhancing the effect of feature extraction. Additionally, YOLOv8 has undergone significant reforms in its head structure, introducing the currently mainstream decoupled head structure and shifting from an anchor-based design to an anchor-free approach, which helps simplify the training process and enhance the model's versatility. In its data augmentation strategy, YOLOv8 has adopted practices from YOLOX, particularly in disabling Mosaic enhancement in the last 10 epochs of training, a strategy proven to effectively improve the model's accuracy. For our model, these improvements in YOLOv8 have significantly enhanced the accuracy and efficiency of detecting brain tumors in MRI images, providing strong technical support for the early diagnosis and precise treatment of brain tumors and showcasing the potential of deep learning in the field of advanced medical image processing. The network architecture of YOLOv8 is shown in Figure 2.

However, YOLOv8 also has some shortcomings. Firstly, traditional convolutional networks may not be flexible enough when dealing with highly heterogeneous medical images, and fixed convolutional kernels may struggle to adapt to complex image features. Secondly, although the basic YOLOv8 model performs well with images that have complex backgrounds, there is still room for improvement in sensitivity and precision for specific medical images, such as MRI. Additionally, the standard loss functions may be inadequate for precise boundary localization of objects within medical images, especially in cases of unclear boundaries or partial obstructions.

In the following sections, we will detail the improvements made to address the aforementioned issues. These enhancements are designed to increase the flexibility, accuracy, and efficiency of the YOLOv8 model in processing medical images, particularly in the detection of brain tumors in MRI scans. Through these innovative approaches, we aim to significantly enhance the model's performance in medical applications, especially in handling brain tumor images with complex backgrounds and unclear boundaries.

### 3.3 KernelWarehouse

**Figure 3:**
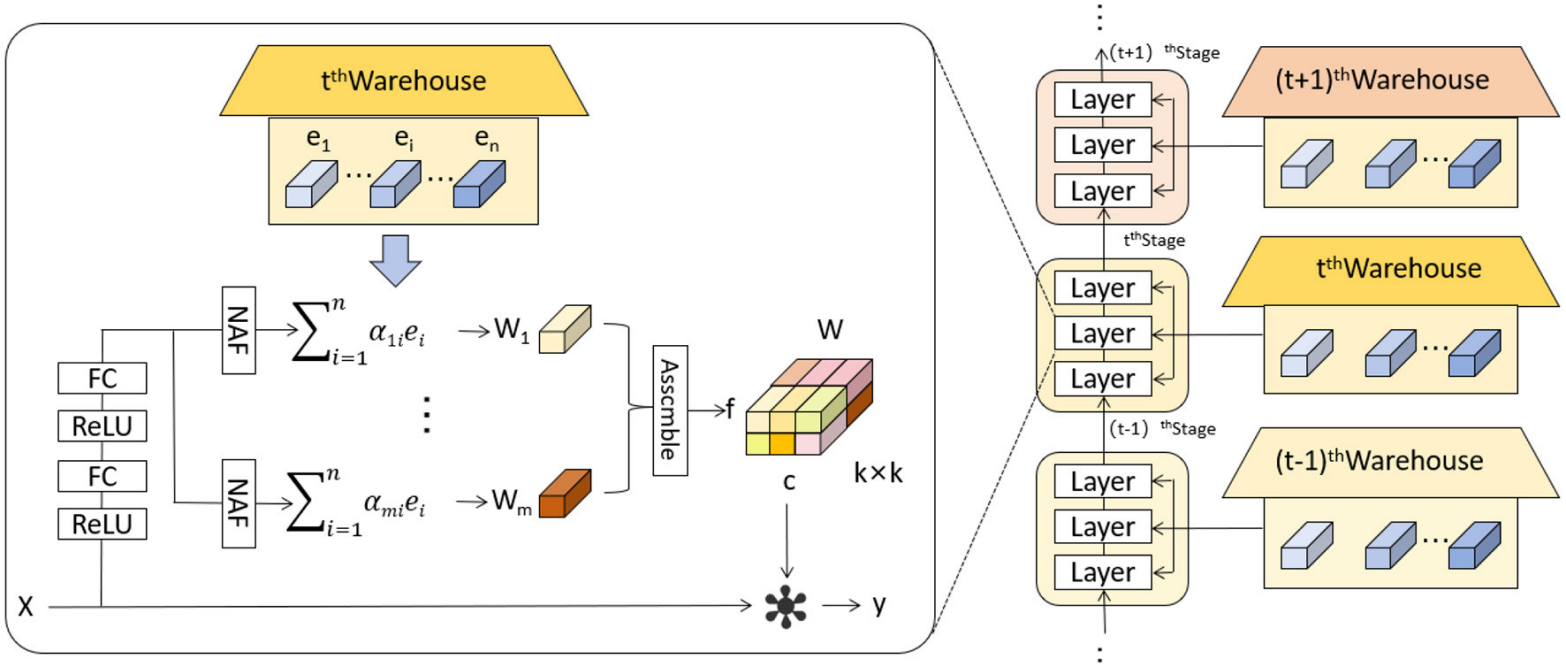
Schematic illustration of KernelWarehouse (36).

The KernelWarehouse model introduces an innovative dynamic convolution approach that enhances the capability of convolutional neural networks to process complex images or sequence data (36). In KernelWarehouse, the standard convolution operation, which typically relies on fixed kernels *W*, is replaced by a flexible kernel mixing mechanism. Specifically, both the input *x* and the output *y* maintain the same spatial resolution, where *x* represents the input features and *y* the output features, while the kernel *W* is no longer static but is a linear combination of multiple static kernels *W* = α_1_*W*_1_ + … + α_*n*_*W*_*n*_, with α_1_, …, α_*n*_ being attention weights based on the input. Figure 3 shows the network architecture of KernelWarehouse. This design of dynamic convolution kernels allows KernelWarehouse to dynamically adjust its processing core based on the features of the input data, achieving more precise feature extraction. Compared to traditional dynamic convolution methods, the innovation of KernelWarehouse lies in its application of the attention-mixing learning paradigm at a more granular kernel level.

Below we introduce the key idea of KernelWarehouse.

The fundamental concept of kernel partitioning is to exploit the dependencies of parameters within the same convolutional layer explicitly. This approach aims to reduce the dimensionality of each kernel while increasing the total number of kernels. The kernel partitioning can be mathematically defined as follows:


(1) $$\begin{array}{l} W=w_{1}\cup \ldots \cup w_{m},\text{ and for all }i,j\in \left\{1,\ldots ,m\right\},i\neq j,w_{i}\cap w_{j}=\emptyset . \end{array}$$

Where *W*: Represents the entire set of convolutional kernels. *w*_*i*_: Represents a subset of the kernel set *W*, *i* indexes the subset. *m*: Indicates the total number of subsets into which *W* is divided. Through this kernel partitioning method, *KernelWarehouse* is able to decompose a large set of convolutional kernels into smaller, independent segments, each focusing on capturing specific features of the input data. This strategy not only enhances the model's ability to process specific data features but also optimizes overall network parameter efficiency and learning outcomes.

Following the straightforward design principle of kernel partitioning, the main goal of warehouse sharing in the KernelWarehouse model is to enhance parameter efficiency and representation capability by explicitly exploiting the dependencies between parameters across consecutive convolutional layers. This approach allows for a more effective sharing and utilization of kernel subsets, optimizing the network's performance by leveraging learned features across different layers.


(2) $$\begin{array}{l} S=s_{1}\cup \ldots \cup s_{n},\text{ where }s_{i}\subset W,\text{ for all }i\in \left\{1,\ldots ,n\right\}. \end{array}$$

Where *S*, which represents a set of kernel subsets shared across different convolutional layers, with each subset *s*_*i*_ being part of the entire kernel set *W*.


(3) $$\begin{array}{l} s_{i}\cap s_{j}=\emptyset ,\text{ for all distinct }i,j\in \left\{1,\ldots ,n\right\}. \end{array}$$

Where *s*_*i*_ is mutually exclusive from others, maintaining independence between different shared partitions.


(4) $$\begin{array}{l} W^{\prime }=\overset{n}{\underset{i=1}{⋃}}\beta_{i}s_{i},\text{ with }\beta_{i}\text{ being the tuning coefficients}. \end{array}$$

Where *s*_*i*_ are combined to form a new effective kernel *W*′ used in the convolution operations. The coefficients β_*i*_ allow for dynamic adjustment of the influence each subset has, which can be tuned based on specific tasks or data characteristics.

### 3.4 Convolutional Block Attention Module

CBAM (Convolutional Block Attention Module) is an advanced attention mechanism designed to enhance the effectiveness of convolutional neural networks by focusing on relevant features within the input data (37). It incorporates attention across both spatial and channel dimensions, ensuring that the network prioritizes the most informative parts of the input.

**Figure 4:**
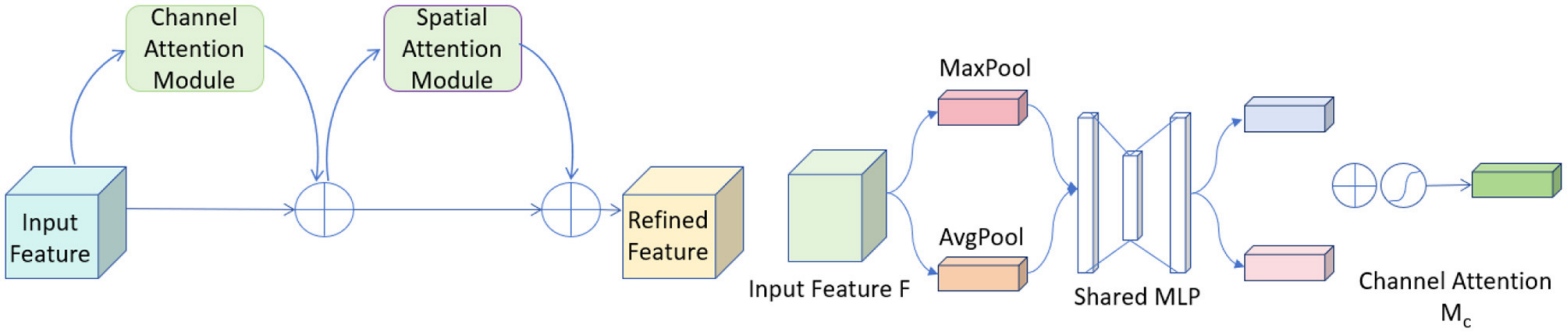
The diagram of the CBAM network structure.

The CBAM module consists of two sequential components: the Channel Attention Module (CAM) and the Spatial Attention Module (SAM). The CAM focuses on identifying the most informative channels. It achieves this by aggregating spatial information through global average pooling and max pooling operations, which emphasize significant channels by exploiting inter-channel relationships. Following this, the SAM directs the network's focus to important spatial locations in the input data. This sequential attention to both channels and spatial locations ensures that the network adapts dynamically to focus more on salient features that are crucial for the task at hand. Figure 4 shows the network architecture diagram of CBAM.

In our model, the integration of CBAM has significantly contributed to improving the overall accuracy and robustness. By allowing the network to focus on the most relevant features and regions, CBAM helps in reducing the influence of irrelevant information and enhances the model's ability to generalize across different and challenging datasets. This makes it particularly valuable in applications such as medical imaging and object detection, where precision is critical. The inclusion of CBAM in our architecture has demonstrably enhanced feature representation capabilities, leading to better performance and more efficient learning. Below, we introduce the mathematical formulas of the CBAM:

First, we compute the channel attention map by combining the features processed through two different pooling strategies:


(5) $$\begin{array}{l} M_{c}=\sigma \left(MLP\left(AvgPool\left(F\right)\right)+MLP\left(MaxPool\left(F\right)\right)\right) \end{array}$$

Where *M*_*c*_ represents the channel attention map, σ is the sigmoid function, *MLP* denotes a multi-layer perceptron, *AvgPool* and *MaxPool* are average and max pooling operations respectively, and *F* is the input feature map.

Next, we apply the channel attention map to the input feature map to enhance relevant channels:


(6) $$\begin{array}{l} F^{\prime }=M_{c}\cdot F \end{array}$$

Where *F*′ is the feature map after applying channel attention, *M*_*c*_ is the channel attention map from the previous equation, and · denotes element-wise multiplication. Following channel attention, we compute the spatial attention map, which highlights important spatial regions using a convolutional filter applied to pooled features:


(7) $$\begin{array}{l} M_{s}=\sigma \left(f^{7\times 7}\left(\left[AvgPool\left(F^{\prime }\right);MaxPool\left(F^{\prime }\right)\right]\right)\right) \end{array}$$

Where *M*_*s*_ is the spatial attention map, σ is the sigmoid function, *f*^7×7^ represents a convolution operation with a 7 × 7 filter, and *AvgPool*, *MaxPool* operations are applied across the channel dimension of *F*′.

The spatial attention map is then applied to the previously refined feature map to further highlight important spatial features:


(8) $$\begin{array}{l} F^{\prime \prime }=M_{s}\cdot F^{\prime } \end{array}$$

Where *F*″ is the output feature map after applying spatial attention, *M*_*s*_ is the spatial attention map from the previous equation, and · denotes element-wise multiplication.

Finally, we combine the attentively processed feature map with the original input to ensure information continuity and completeness:


(9) $$\begin{array}{l} \hat{F}=F^{\prime \prime }+F \end{array}$$

Where $\hat{F}$ is the final output feature map, *F*″ is the feature map after applying spatial attention, and *F* is the original input feature map, demonstrating the residual connection.

### 3.5 Inner-GIoU

**Figure 5:**
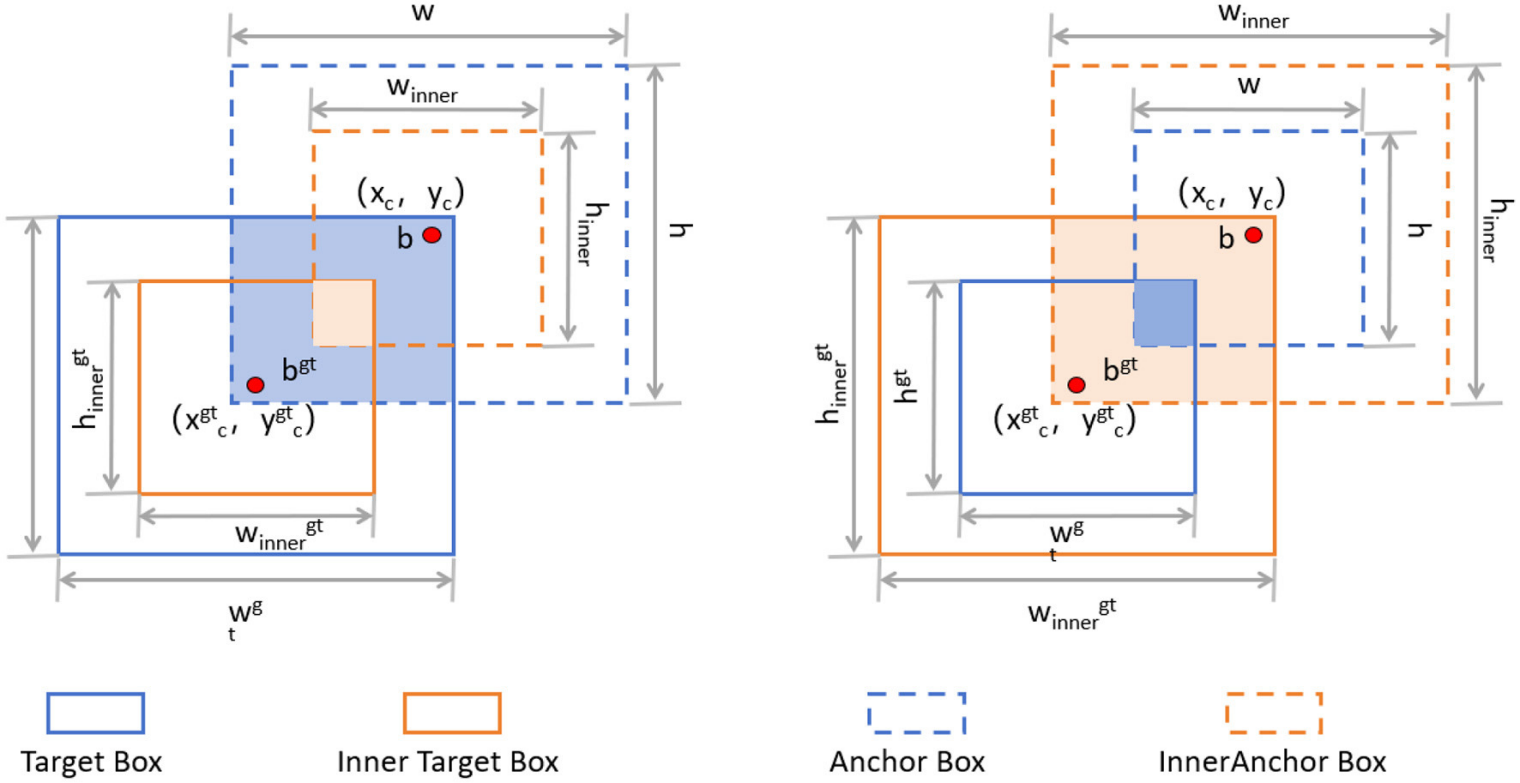
Description of Inner-IoU (38).

The Inner-IoU loss function is an innovative approach designed to address some inherent limitations in traditional IoU (Intersection over Union) loss calculations (38), especially in bounding box regression (BBR) tasks like those in YOLOv8. Traditional IoU-based BBR methods often accelerate convergence by adding new loss components but tend to overlook the limitations of the IoU loss itself. The Inner-IoU loss function starts with an in-depth analysis of the BBR model, revealing that differentiating between various regression samples and using auxiliary bounding boxes of different scales for loss computation can effectively speed up the bounding box regression process. Specifically, for samples with high IoU, using smaller auxiliary bounding boxes to compute the loss can accelerate convergence; whereas for low IoU samples, larger auxiliary bounding boxes are more appropriate. By introducing a scaling factor ratio to control the scale of the auxiliary bounding boxes used for loss computation, the bounding box regression process is optimized. Figure 5 illustrates the concept of Inner-IoU.

The introduction of the Inner-IoU loss function has significantly enhanced the accuracy and efficiency of our model in detecting brain tumors in MRI images. Through a refined loss calculation strategy, our model can adjust bounding boxes more quickly and accurately, thereby improving the robustness and precision of detection. The incorporation of the scaling factor ratio allows the model to dynamically adjust the loss calculation method when handling different IoU samples, resulting in greater adaptability and exceptional performance in various complex scenarios. Below, we introduce the main mathematical derivation process of Inner-IoU. IoU is defined as follows:


(10) $$\begin{array}{l} IoU=\frac{\left|B\cap B^{gt}\right|}{\left|B\cup B^{gt}\right|} \end{array}$$

Where *B* and *B*^*gt*^ represent the predicted box and the ground truth (GT) box, respectively. Additionally, GIoU was developed to address the problem of gradient vanishing when the anchor box and the GT box have zero overlap. The GIoU loss function is defined as follows:


(11) $$\begin{array}{l} L_{GIoU}=1-IoU+\frac{\left|C-B\cap B^{gt}\right|}{\left|C\right|} \end{array}$$

To derive Inner-IoU in more detail, we need to understand the following concepts. The ground truth (GT) box and anchor are denoted as *B*_*gt*_ and *B*, respectively. The center points are (*x*_*gtc*_, *y*_*gtc*_) for the GT box and (*x*_*c*_, *y*_*c*_) for the anchor. The width and height of the GT box are *w*_*gt*_ and *h*_*gt*_, while those of the anchor are *w* and *h*. The variable “ratio” is the scaling factor, typically between [0.5, 1.5]. The specific expressions are as follows:


(12) $$\begin{array}{l} b_{l}^{gt}=x_{c}^{gt}-\frac{w_{gt}*ratio}{2},\;b_{r}^{gt}=x_{c}^{gt}+\frac{w_{gt}*ratio}{2} \end{array}$$

(13) $$\begin{array}{l} b_{t}^{gt}=y_{c}^{gt}-\frac{h_{gt}*ratio}{2},\;b_{b}^{gt}=y_{c}^{gt}+\frac{h_{gt}*ratio}{2} \end{array}$$

(14) $$\begin{array}{l} b_{l}=x_{c}-\frac{w*ratio}{2},\;b_{r}=x_{c}+\frac{w*ratio}{2} \end{array}$$

(15) $$\begin{array}{l} b_{t}=y_{c}-\frac{h*ratio}{2},\;b_{b}=y_{c}+\frac{h*ratio}{2} \end{array}$$

(16) $$\begin{array}{l} inter=\left(min\left(b_{r}^{gt},b_{r}\right)-max\left(b_{l}^{gt},b_{l}\right)\right)*\left(min\left(b_{b}^{gt},b_{b}\right)-max\left(b_{t}^{gt},b_{t}\right)\right) \end{array}$$

(17) $$\begin{array}{l} union=\left(w_{gt}*h_{gt}\right)*{\left(ratio\right)}^{2}+\left(w*h\right)*{\left(ratio\right)}^{2}-inter \end{array}$$

The final formula for Inner-IoU is as follows:


(18) $$\begin{array}{l} IoU_{inner}=\frac{inter}{union} \end{array}$$

Through extensive experiments, we finally combined Inner-IoU with GIoU, resulting in the final loss function as follows:


(19) $$\begin{array}{l} L_{Inner-GIoU}=L_{GIoU}+IoU-IoU_{inner} \end{array}$$

Where *L*_Inner-GIoU_ represents the Inner-GIoU loss proposed in this paper, *IoU*_inner_ represents Inner-IoU, and *L*_GIoU_ represents the GIoU loss. By incorporating KernelWarehouse, CBAM, and Inner-GIoU, we successfully enhanced the accuracy and efficiency of brain tumor detection in MRI images. KernelWarehouse optimized the flexibility of convolutional kernels, CBAM improved feature extraction capabilities, and Inner-GIoU refined the loss function calculation. These innovations combined to enable our model to excel in processing complex medical images, providing stronger technical support for clinical diagnosis.

## 4 Experiments

### 4.1 Datasets

**Figure 6:**
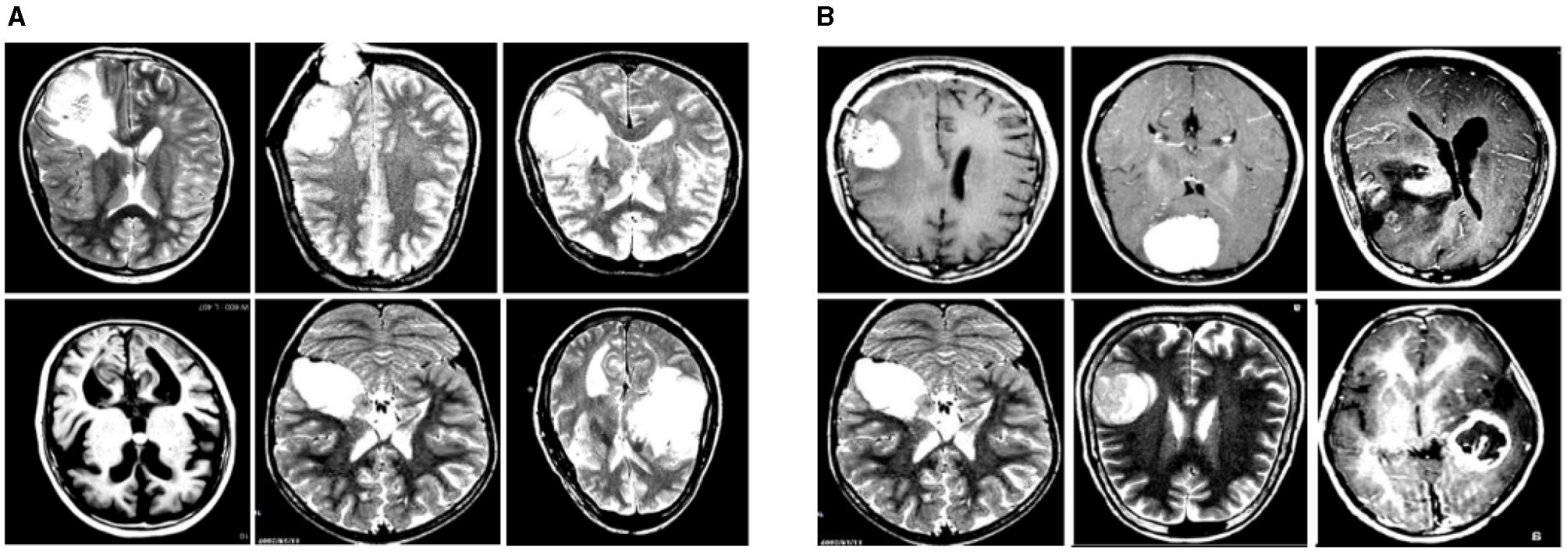
Sample displays of the Br35H dataset and the Roboflow dataset. **(A)** shows a sample from the Br35H dataset. **(B)** shows a sample from the Roboflow dataset.

In this experiment, we used two datasets to validate the effectiveness of our method: the Br35H dataset (39) and the open-source Roboflow dataset (40). Figure 6 shows samples from these datasets.

The Br35H dataset is an MRI image dataset for brain tumor detection and classification, containing 801 brain MRI images. These images are divided into three parts: 561 images for training, 160 images for testing, and 80 images for validation. The images are classified into categories of with tumor and without tumor. The Br35H dataset provides a moderate amount of data and diversity for deep learning-based brain tumor detection and classification.

The Roboflow dataset is an open-source brain tumor MRI image dataset, containing a total of 76 images. For the experiment, the dataset is divided as follows: 60 images for training, nine images for testing, and seven images for validation. The Roboflow dataset also includes MRI images classified as with tumor and without tumor, providing a rich data source for our experiments.

Table 1 shows the specific division of the datasets. Through experiments on these two datasets, we can comprehensively validate the effectiveness and robustness of our method under different data conditions. The Br35H dataset offers a larger data volume suitable for training and validating deep learning models, while the Roboflow dataset provides additional diversity and challenges through its smaller scale, ensuring our method performs well under various conditions. This dual-validation strategy makes our research results more reliable and widely applicable.

**Table 1:** The data set partitioning is in the Br35H dataset and the Roboflow dataset.

**Dataset**	**Training set**	**Testing set**	**Validation set**
Br35H	561	160	80
Roboflow	60	9	7

### 4.2 Experiments environment

The hardware and software configurations for this experiment are shown in Table 2. It includes a computer equipped with an Intel(R) Xeon(R) Gold 6129 CPU, running at a frequency of 2.30 GHz with 32 cores. The system also features ten NVIDIA Tesla V100-PCIE GPUs, totaling 160GB of VRAM, and 187GB of memory, ensuring ample computational resources to handle complex tasks.

**Table 2:** Experiment configuration environment.

**Configuration**	**Name**	**Specific information**
Hardware environment	CPU	Intel(R) Xeon(R) Gold 6129 CPU @ 2.30G Hz*32
	GPU	NVIDIA Tesla V100-PCIE*10
	VRAM	160 GB
	Memory	187 GB
Software environment	Operating system	Ubuntu
	Python version	3.9.18
	PyTorch version	1.13.0
	CUDA version	11.3
	OpenCV version	4.6.0

### 4.3 Metrics

In this experiment, our method evaluates the performance of the model using the following metrics: Precision (PR), Recall (RE), Sensitivity (SE), Specificity (SP), Accuracy (AC), F1-Score, and mAP50. Below, we detail these metrics and provide the corresponding formulas.

Precision (PR) measures the proportion of true positive samples among the samples predicted as positive by the model. The formula is:


(20) $$\begin{array}{l} PR=\frac{TP}{TP+FP} \end{array}$$

where TP (True Positive) represents the true positives, and FP (False Positive) represents the false positives.

Recall (RE) measures the proportion of actual positive samples correctly predicted as positive by the model. The formula is:


(21) $$\begin{array}{l} RE=\frac{TP}{TP+FN} \end{array}$$

where FN (False Negative) represents the false negatives.

Sensitivity (SE) is synonymous with recall, measuring the proportion of actual positive samples correctly predicted as positive by the model. The formula is:


(22) $$\begin{array}{l} SE=\frac{TP}{TP+FN} \end{array}$$

Specificity (SP) measures the proportion of actual negative samples correctly predicted as negative by the model. The formula is:


(23) $$\begin{array}{l} SP=\frac{TN}{TN+FP} \end{array}$$

where TN (True Negative) represents the true negatives.

Accuracy (AC) measures the proportion of correct predictions among all predictions made by the model. The formula is:


(24) $$\begin{array}{l} AC=\frac{TP+TN}{TP+TN+FP+FN} \end{array}$$

F1-Score is the harmonic mean of precision and recall, providing a balance between the two metrics. The formula is:


(25) $$\begin{array}{l} F1=2\cdot \frac{PR\cdot RE}{PR+RE} \end{array}$$

mAP calculates the mean of average precisions across all classes:


(26) $$\begin{array}{l} mAP=\frac{1}{N}\overset{N}{\underset{i=1}{\sum }}AP_{i} \end{array}$$

Where *AP*_*i*_ is the average precision for the *i*^*th*^ class and *N* is the total number of classes.

Through these metrics, we can comprehensively evaluate the performance of the model on different datasets. These metrics help us understand the model's performance and reliability in various classification tasks.

### 4.4 Implementation details

#### 4.4.1 Parameter settings

In this experiment, we carefully set the model parameters to optimize the training process and performance. The specific parameters include: an initial learning rate set to 0.001, with a cosine annealing scheduler gradually reducing the learning rate to ensure stable convergence in the later stages of training; a batch size of 16 to ensure efficient use of computational resources and accelerate the training process; 300 epochs to ensure the model fully learns the data features and improves generalization ability; a weight decay coefficient of 0.0005 to prevent overfitting; the Adam optimizer, which combines momentum and adaptive learning rates to effectively speed up model convergence; the model consists of 245 layers with a total of 10,236,528 parameters, ensuring sufficient complexity to capture subtle features in MRI images. The specific settings are shown in Table 3.

**Table 3:** Training parameters.

**Parameter**	**Value**
Learning Rate	0.001
Batch Size	16
Weight Decay	0.0005
Epochs	300
Layers	245
Parameters	10,236,528

#### 4.4.2 Algorithm process

Algorithm 1 illustrates the training process of our network, using the Br35H dataset and the open-source Roboflow dataset. First, the model parameters and hyperparameters are initialized. Then, for each training epoch, forward propagation, loss calculation, and gradient updates are performed for each batch. We introduced the Inner-GIoU loss function to improve the accuracy of bounding box localization. The model's performance is continuously evaluated on the validation set, and if performance improves, the model parameters are saved, ensuring the model's efficiency and accuracy under different data conditions.

**Algorithm 1:**
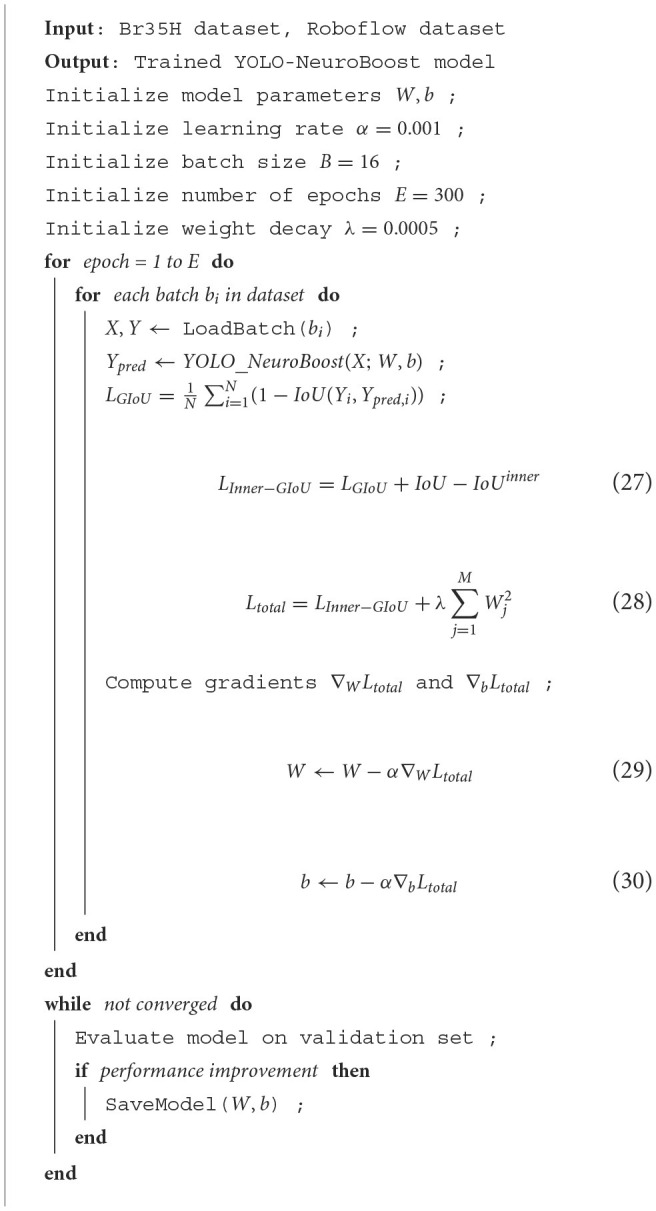
Training YOLO-NeuroBoost on Br35H and Roboflow Datasets

#### 4.4.3 Training results

**Figure 7:**
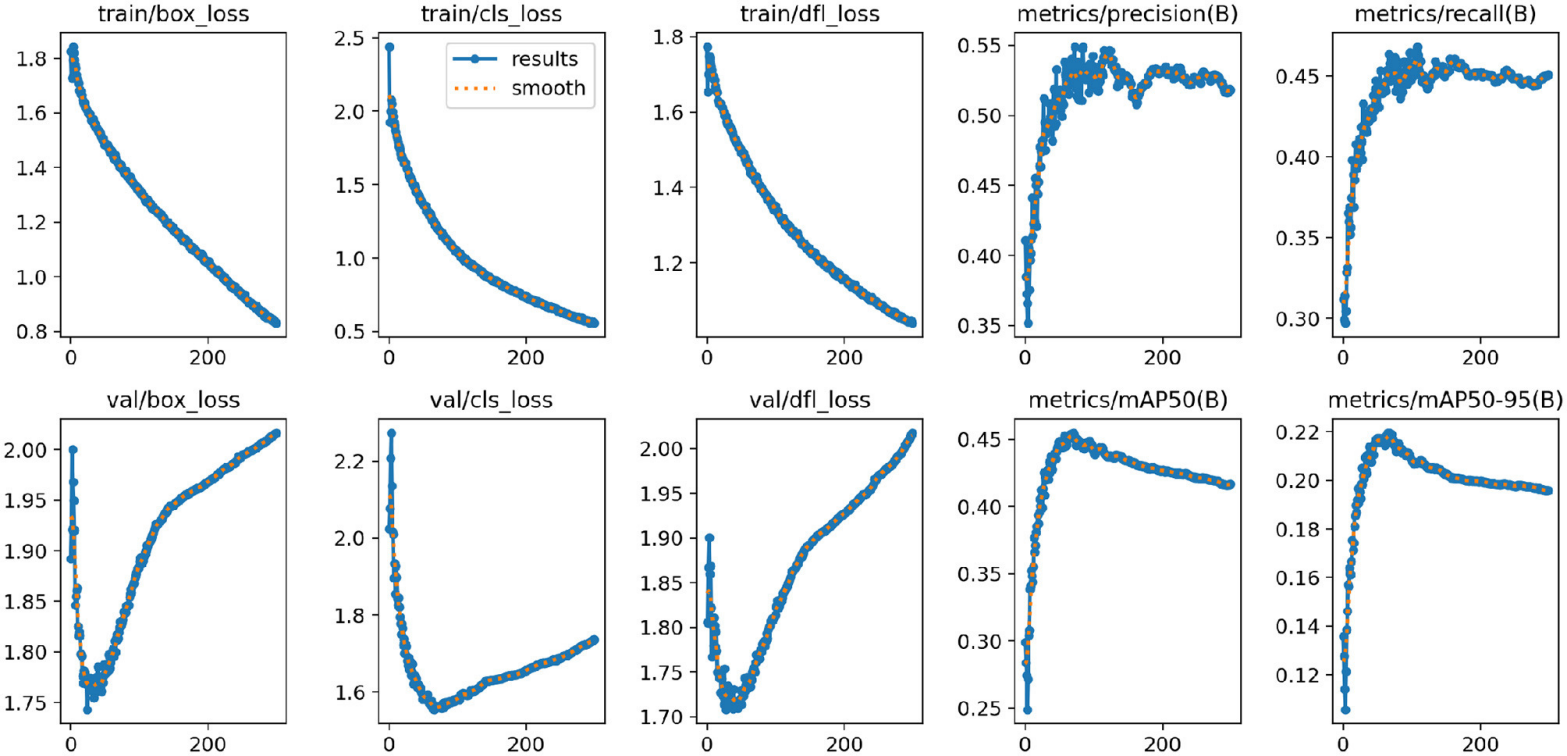
The results of the proposed model.

Figure 7 shows the changes in the loss functions and evaluation metrics during the training and validation process of the YOLO-NeuroBoost model. The figure includes the trends of the bounding box loss, classification loss, and dynamic convolution loss on both the training and validation sets, demonstrating that these losses gradually decrease as training progresses. Additionally, the figure shows the improvement trends in metrics such as Precision, Recall, mAP50, and mAP50-95. These results indicate that our model effectively improves the accuracy and robustness of tumor detection in brain MRI images, highlighting the strong potential of YOLO-NeuroBoost in the field of medical image analysis.

### 4.5 Comparison to prior work

As shown in Table 4, the experimental section presents a comparison of the performance of different models on the Roboflow and Br35H datasets. We evaluated each model's performance across several key performance metrics, including mean Average Precision (mAP50), Precision Rate (PR), Recall (AC), and F1-Score. The YOLO-NeuroBoost model demonstrated its superior performance on the Roboflow dataset, leading in all metrics. Specifically, the model achieved an mAP50 of 97.71%, PR of 98.67%, AC of 97.72%, and an F1-Score of 97.63%. Although other models, such as YOLOv8n, performed slightly less impressively with an mAP50 of 95.92%, this still reflects a relatively high level of accuracy. In the Br35H dataset, the advantages of the YOLO-NeuroBoost model were even more apparent, as it significantly outperformed other models across all assessment metrics. Specific performances included an mAP50 of 99.52%, PR of 99.48%, AC of 99.51%, and an F1-Score of 99.45%. While other models also performed well on this dataset, they were significantly behind YOLO-NeuroBoost.

**Table 4:** Comparison of model performance on Roboflow and Br35H datasets.

	**Roboflow dataset**				**Br35H dataset**			
**Models**	**mAP50 (%)**	**PR (%)**	**AC (%)**	**F1-Score (%)**	**mAP50 (%)**	**PR (%)**	**AC (%)**	**F1-Score (%)**
YOLOv4 (32)	93.91	93.67	93.87	93.03	95.33	95.47	95.649	95.85
SSD (41)	94.47	94.37	94.55	94.68	96.25	96.17	96.34	96.42
YOLOv7-Tiny (42)	94.85	94.45	94.77	94.94	96.94	96.24	96.54	96.78
VitDet (29)	94.97	94.54	94.67	94.17	96.71	96.35	96.48	96.92
YOLOv5n (33)	95.67	95.57	95.87	95.77	97.45	97.35	97.63	97.53
YOLOv8n (43)	95.92	95.76	96.07	96.07	97.72	97.56	97.82	97.82
YOLO-NeuroBoost	**97.71**	**98.67**	**97.72**	**97.63**	**99.52**	**99.48**	**99.51**	**99.45**

Bold indicates the best results.

YOLO-NeuroBoost demonstrated optimal performance on two datasets with distinct characteristics. This not only validates the model's efficiency but also emphasizes its adaptability to different data distributions and significant robustness. Such robustness indicates that YOLO-NeuroBoost has the capability to maintain stable performance in varying environments, providing a solid foundation for its widespread use in tumor detection and other complex application areas.

**Figure 8:**
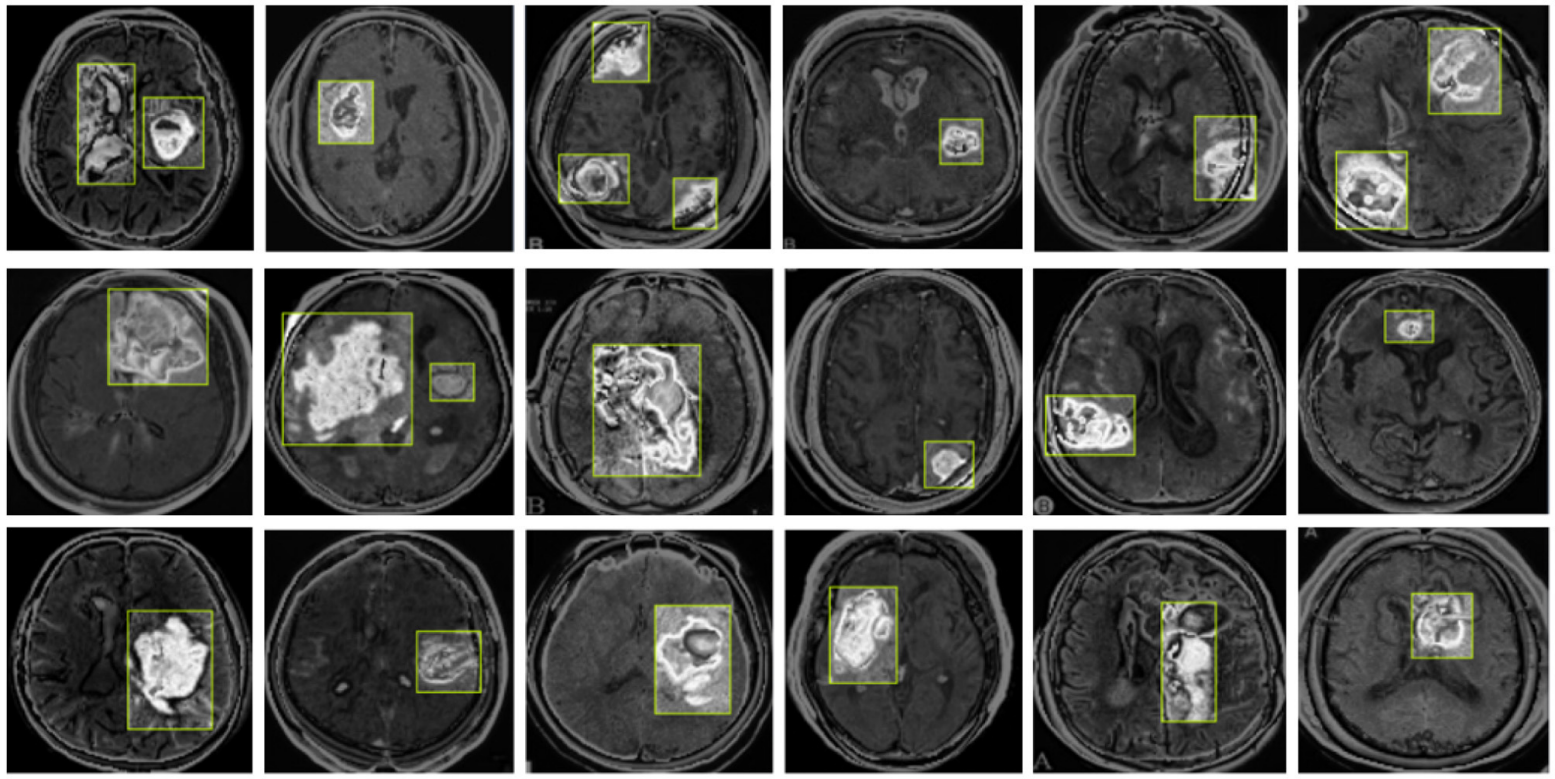
YOLO-NeuroBoost brain tumor detection visualization.

Figure 8 illustrates the detection results of YOLO-NeuroBoost, demonstrating its clear advantage in the task of brain tumor detection in MRI images. This further validates the outstanding performance of the YOLO-NeuroBoost model across different datasets, confirming its effectiveness and generalization capability.

**Figure 9:**
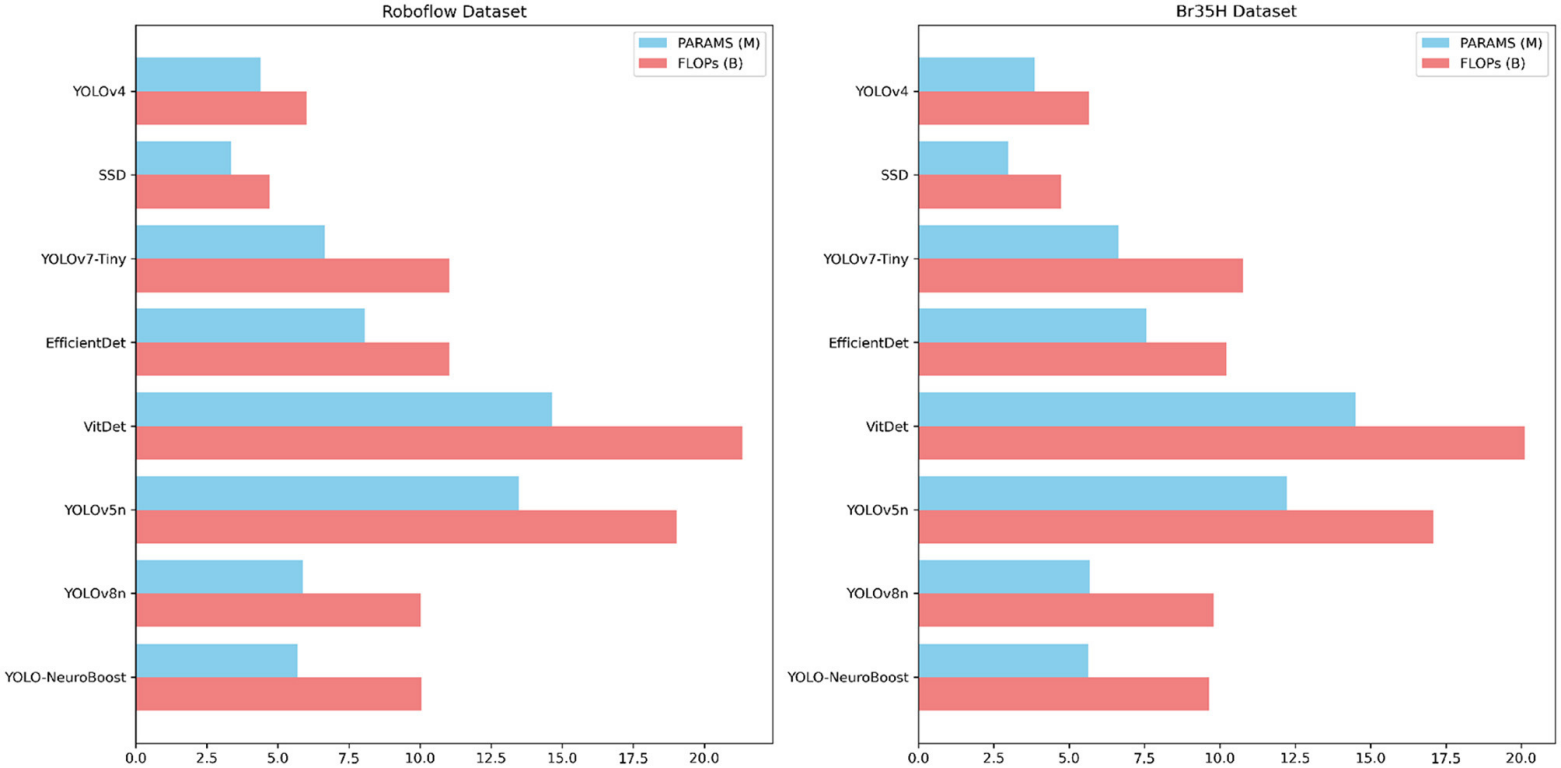
Different model parameter comparisons.

Table 5 compares the PARAMS and FLOPs of various models on the Roboflow and Br35H datasets, with the YOLO-NeuroBoost algorithm demonstrating outstanding performance. Compared to other models, YOLO-NeuroBoost exhibits lower PARAMS and FLOPs on both datasets, yet delivers remarkable performance, comparable to models with higher parameter counts and computational loads. On the Roboflow dataset, YOLO-NeuroBoost has 5.68 million parameters and performs 10.03 billion floating-point operations, while on the Br35H dataset, it has 5.65 million parameters and 9.65 billion floating-point operations. This indicates that YOLO-NeuroBoost maintains excellent performance while reducing model complexity, thus providing higher efficiency and feasibility for practical applications. Figure 9 visualizes these data.

**Table 5:** Comparison of model parameters (PARAMS) and floating point operations (FLOPs) on Roboflow and Br35H datasets.

**Model**	**Roboflow dataset**		**Br35H dataset**	
	**PARAMS**	**FLOPs**	**PARAMS**	**FLOPs**
YOLOv4	4.38 M	6.01 B	3.86 M	5.67 B
SSD	3.35 M	4.72 B	2.98 M	4.73 B
YOLOv7-Tiny	6.65 M	11.03 B	6.65 M	10.78 B
EfficientDet	8.07 M	11.03 B	7.56 M	10.21 B
VitDet	14.66 M	21.34 B	14.51 M	20.12 B
YOLOv5n	13.48 M	19.02 B	12.23 M	17.08 B
YOLOv8n	5.88 M	10.01 B	5.68 M	9.81 B
YOLO-NeuroBoost	5.68 M	10.03 B	5.65 M	9.65 B

### 4.6 Ablation study

Table 6 presents the results of ablative experiments on various components. We evaluated different configurations of the YOLO-NeuroBoost model and classified them based on whether they include the KernelWarehouse, CBAM, and Inner-GIoU components. Evaluation metrics include mAP50 and mAP50-95. Each row represents a model variant, with a checkmark indicating inclusion and a cross indicating exclusion of components. Firstly, the YOLOv8n model performs at a baseline level when all components are absent (YOLOv8n), with an mAP50 of 83.26. Then, introducing the KernelWarehouse (YOLOv8n-1), CBAM (YOLOv8n-2), and Inner-GIoU (YOLOv8n-3) components individually slightly improves performance, with limited improvement, yielding mAP50 scores of 85.32, 83.26, and 85.34 respectively. Next, simultaneously introducing the KernelWarehouse and CBAM components (YOLOv8n-4) significantly boosts model performance, with an mAP50 of 90.87, indicating a significant impact of combining these two components. Furthermore, introducing all three components into the YOLOv8n model (YOLOv8n-5) results in even greater performance improvement, with an mAP50 of 95.33, demonstrating the additional effect of the Inner-GIoU component. Finally, incorporating all three components into the YOLO-NeuroBoost model (YOLO-NeuroBoost) achieves the highest performance level, with an mAP50 of 97.71, showcasing the advantage of the comprehensive interaction of components.

**Table 6:** Experiment results for each component.

**Method**	**KernelWarehouse**	**CBAM**	**Inner-GIoU**	**mAP50**	**mAP50-95**
YOLOv8n	✗	✗	✗	83.26	85.37
YOLOv8n-1	✓	✗	✗	85.32	84.33
YOLOv8n-2	✗	✓	✗	83.26	85.67
YOLOv8n-3	✗	✗	✓	85.34	82.15
YOLOv8n-4	✓	✓	✗	90.87	87.66
YOLOv8n-5	✗	✓	✓	95.33	92.15
YOLO-NeuroBoost	✓	✓	✓	**97.71**	**95.32**

Bold indicates the best results.

### 4.7 Qualitative Results

#### 4.7.1 Detection of small tumors

**Figure 10:**
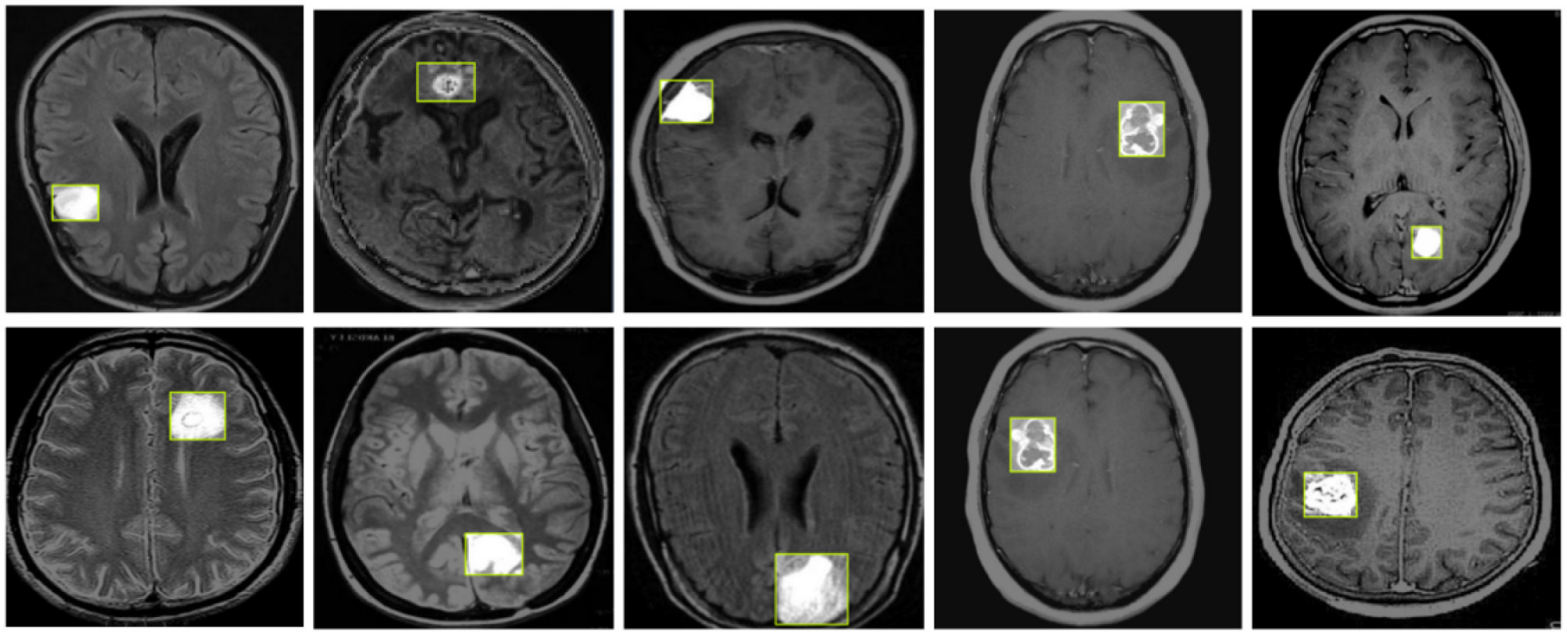
YOLO-NeuroBoost small size brain tumor detection.

Our model also performs well in detecting small tumors. As shown in Figure 10, through testing on tumors of different sizes, we found that the YOLO-NeuroBoost model demonstrates outstanding performance in detecting small-sized targets. Not only does our model excel in detecting large targets, but it also exhibits remarkable robustness and accuracy in detecting small tumors. This capability is particularly crucial in fields like medical image analysis, where small anomalies may be harder to perceive but could have significant clinical implications.

#### 4.7.2 Explainability illustration

**Figure 11:**
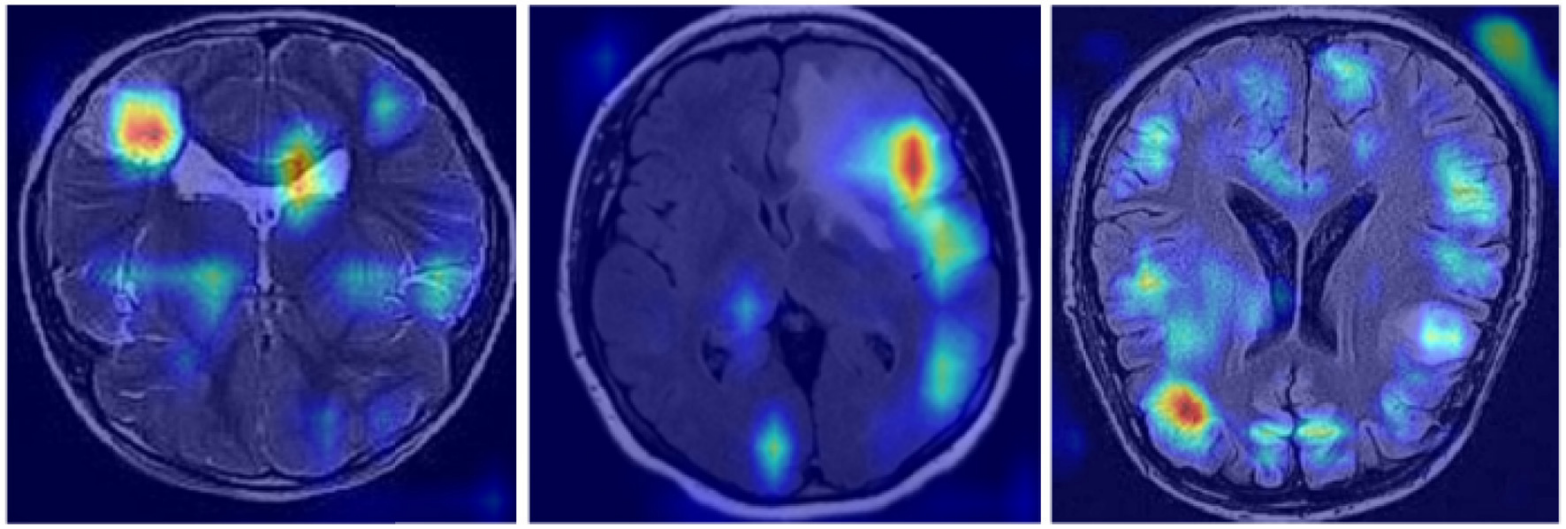
Brain tumor feature explainability display.

In Figure 11, we demonstrate the efficiency and interpretability of our model through feature visualization techniques. Using Grad-CAM (Gradient-weighted Class Activation Mapping), our model is able to highlight key features within the tumor images, clearly presenting these in the visualization results. The model not only shows how it focuses on important areas within the images, such as the boundaries and structural details of abnormal tissues, but also aligns highly with the aspects medical experts consider in tumor diagnosis. This interpretability is extremely important in the medical field, as it allows doctors and specialists to deeply understand the model's decision-making process by analyzing these feature maps, leading to more precise diagnoses. Additionally, these intuitive visual explanations not only enhance trust in the model's decision-making process but also provide valuable diagnostic support in clinical practice.

#### 4.7.3 Classification performance

**Figure 12:**
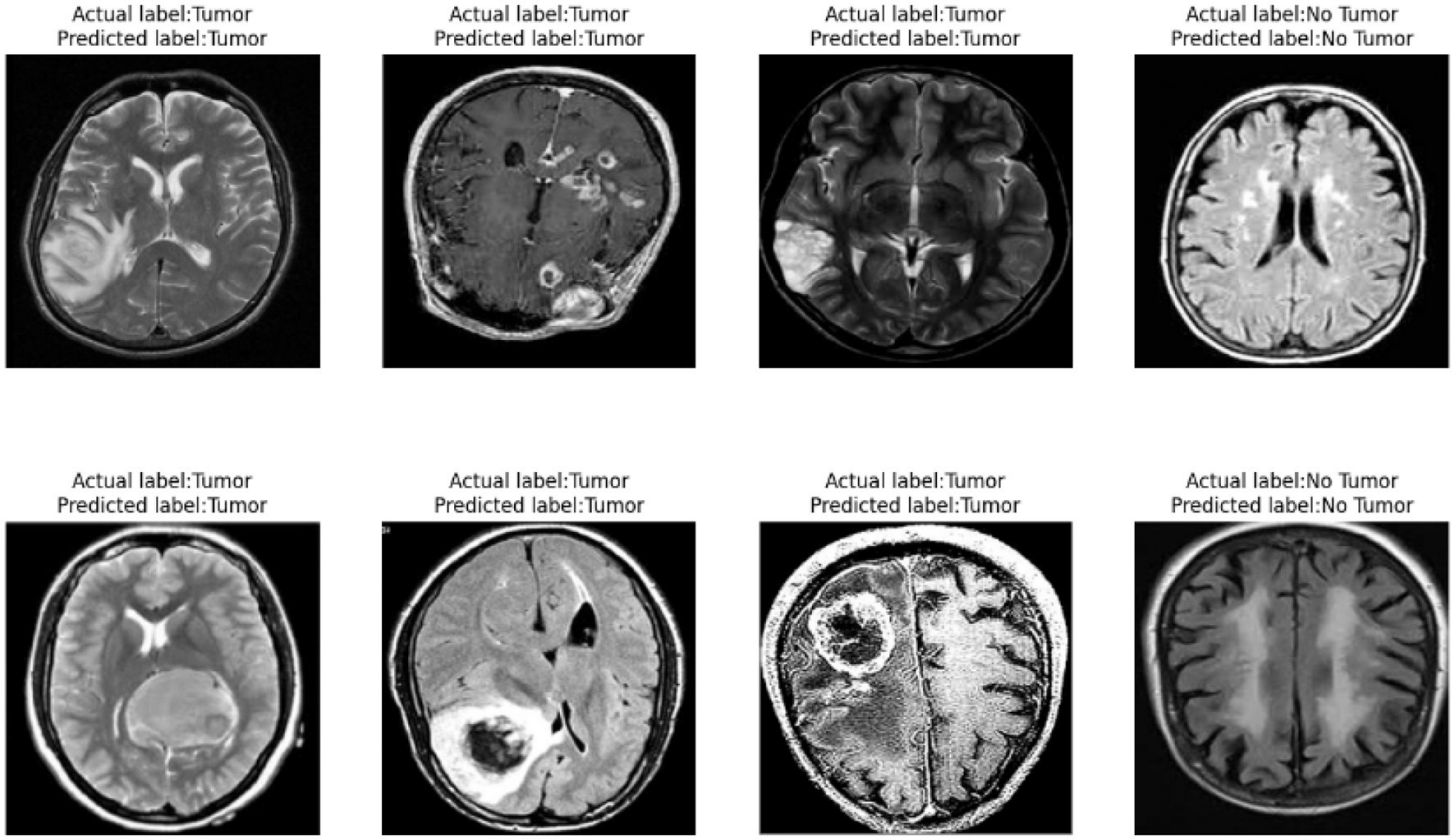
Brain tumor classification performance display.

As shown in Figure 12, our model demonstrates outstanding classification performance. Through extensive training and testing with large datasets, we have validated that the model can accurately classify the presence of tumors under various circumstances. Importantly, our model not only effectively identifies and distinguishes various complex features within tumor images but also exhibits excellent robustness when facing challenging diagnostic scenarios. This remarkable classification capability provides reliable support for our model in real-world applications, meeting diverse requirements in medical diagnosis. It offers substantial assistance to healthcare professionals, enabling more precise and timely treatment decisions, ultimately maximizing patients' chances of recovery and survival.

### 4.8 Discussion

We propose the YOLO-NeuroBoost model, which achieved mAP50 scores of 97.71 and 99.52 on the Roboflow dataset and Br35H dataset, respectively, surpassing the performance of models such as yolov8m. This significant improvement is mainly attributed to the KernelWarehouse convolutional operations. This module uses precise feature extraction techniques, optimizing the model's ability to capture crucial information from images. Compared to traditional dynamic convolution, our method applies attention-based hybrid learning paradigms at a finer kernel level, dynamically adjusting the convolutional kernel responses to achieve adaptive feature extraction, thereby enhancing the model's generalization capability and efficiency. Additionally, for the problem of irrelevant information in MRI datasets, we integrated the CBAM attention mechanism. CBAM, through its spatial and channel attention modules, effectively distinguishes and emphasizes the most critical features for diagnosis in the images while suppressing irrelevant information, further enhancing the model's performance in handling medical imaging data. Finally, we introduced the Inner-GIoU loss calculation method, which improves upon traditional IoU loss by using smaller auxiliary bounding boxes to optimize loss calculation. This strategy not only accelerates the model's convergence speed but also improves the accuracy of target detection in complex backgrounds.

Through ablation experiments, we found that each component plays a critical role in enhancing the model's performance. The inclusion of KernelWarehouse, CBAM, or Inner-GIoU individually results in performance improvements, but not significantly. However, when these components are used in combination, the model's performance is significantly enhanced, especially when all three components are included, where the YOLO-NeuroBoost model outperforms other versions on these datasets. Lastly, Figure 11 presents feature visualizations, where we observed that the model can accurately identify and locate target objects. This provides a reliable basis for future in-depth research and optimization, promising further breakthroughs in MRI tumor image recognition.

## 5 Conclusion

In this study, we introduce an improved YOLOv8 algorithm, termed the YOLO-NeuroBoost model, specifically designed for detecting brain tumors in MRI images. By integrating advanced techniques such as KernelWarehouse, CBAM, and Inner-GIoU, our method significantly enhances the accuracy and robustness of tumor localization and recognition within the images. Despite the exceptional performance of our model in most scenarios, it still encounters challenges in cases with low contrast or high image noise. For our research, we utilized the Br35H and Roboflow datasets, which offer a range of MRI images. However, the diversity of these datasets may still be insufficient to comprehensively represent the various types, scanning parameters, and quality of MRI images. This limitation could potentially affect the model's generalization capabilities and effectiveness in practical applications.

Future endeavors will address several key areas: enhancing image processing techniques to improve robustness against low-quality images; expanding the dataset to include a wider variety of MRI images with varying complexities, thereby boosting the model's generalizability; and exploring methods to more effectively leverage information from multimodal MRI images to elevate model performance. These initiatives are expected to propel significant progress in the medical imaging domain, offering robust support for clinical diagnostics and therapeutic applications.

## Data Availability

The raw data supporting the conclusions of this article will be made available by the authors, without undue reservation.
